# Temperature and composition dependent excitonic luminescence and exciton-phonon coupling in CdSeS nanocrystals

**DOI:** 10.1186/1556-276X-7-301

**Published:** 2012-06-08

**Authors:** Wenzhi Wu, Dongqi Yu, Hong-an Ye, Yachen Gao, Qing Chang

**Affiliations:** 1 School of Electronic Engineering, Heilongjiang University, Harbin 150080, Peoples Republic of China; 2 School of Physics and Electronic Technology, Liaoning Normal University, Dalian, 116029, Peoples Republic of China

**Keywords:** 71.35.Aa, 71.55.Gs, 78.47.jd, 78.67.Hc.

## Abstract

The yellow- and red-emitting CdSeS nanocrystals (NCs) synthesized through one-step organometallic synthesis method are uniformly assembled in polymethyl methacrylate (PMMA). A higher-energy emission band originates from band-edge excitonic state appeared at low temperature. With the Se dopant concentration increasing, the luminescent spectra of CdSeS NCs have a red-shifted emission peak and a shorter luminescent lifetime, which is attributed to the existence of trapping state caused by surface defect and Se dopant. CdSeS NC shows a shorter luminescence lifetime and higher energy emission peak in PMMA matrix than that in toluene, indicating that the former is more favorable to transfer energy through exciton-phonon coupling. The upconversion luminescence (UCL) is observed using 800 nm femtosecond laser excitation. The pump power dependence demonstrated UCL spectra of yellow-emitting CdSeS NCs has a slope of 2.2, while that of red-emitting CdSeS NCs has a slope of 1.4. The results demonstrate that the two-photon absorption plays a dominating role when Se concentration of CdSeS NCs is lower, while phonon-assisted UCL by one-photon excitation gradually takes place with the amount of Se dopants increasing.

## Background

Various methods of synthesizing semiconductor nanocrystals (NCs) have been investigated in liquid phase, in which NCs are suspended in most organic solvents or aqueous solution, making them less practical for fabrication and integration into optoelectronic devices. Therefore, the solidification of NCs in matrix is prerequisite before the assembling of NCs into electronic and optoelectronic devices. Recently, hybrid organic/inorganic nanocomposites have recently attracted considerable interests due to their promising optoelectronic properties and applications. Blends of conjugated polymers and colloidal semiconductor quantum dots have been advantageously used for light-emitting diodes [1-3], ultrasensitive radiation detection [4], and solar energy conversion [5]. To realize flexible color conversion or emitting devices, it is desirable to synthesize composite materials consisting of NCs and polymers, in which the thermally stable semiconductor NCs work as the color-emission centers and the transparent polymers as the embedding matrix materials [6,7]. In addition to this application-driven demand, fundamental physical questions on the coupling between Frenkel excitons in organic molecules and Wannier excitons in inorganic semiconductors have gained enormous attentions [8].

The alloyed and doped NCs are promising in biological and luminescent bifunctional applications [9-11]. In previous works, the synthesis, basic optical properties, and structural characteristics of doped CdSeS NCs have been reported [12,13], but the time-resolved luminescent property is lack of investigation. In this work, we describe a simple prepolymerization method to prepare doped NCs/polymethyl methacrylate (PMMA) composite materials with uniform distribution. Taking into account that exciton luminescence is a strongly temperature-dependent process, it should be possible to distinguish mechanism of radiative and nonradiative transition in NCs/PMMA composite materials. Then, the luminescent mechanisms of doped CdS NCs in different host materials are investigated using time-resolved luminescence technique.

## Methods

In the synthesis of CdSeS NCs, Cd, Se, and S, precursor solutions are prepared separately in three necked flasks. A mixture of CdO, oleic acid, and 1-octadecene is heated at 300°C to get a clear solution used as the Cd precursor. The selenium and sulfur source mixture with different molar ratios in trioctylphosphine is prepared and injected into the hot precursor reaction medium of CdO in solution [14,15]. The reaction solutions are injected into methanol and then CdSeS NCs powder is generated after evaporation of organic solvent. The dopant incorporation into semiconductors NCs plays an important role in optical and structural characteristics [12]. Doped CdSeS NCs are prepared in TOPO solvent. The Se/S molar ratios are 0.04:1 and 0.10:1 for yellow- and red-emitting NCs, respectively, which are revealed by induced coupled plasma mass spectroscopy. For simplicity, CdSeS NCs with molar ratio (0.04:1) emit yellow light, which are called yellow-emitting NCs; CdSeS NCs with molar ratio (0.10:1) emit red light, which are called red-emitting NCs. The yellow- and red-emitting NCs are dispersed in toluene at a concentration of 1 mg/mL. Under vigorous stirring, 0.2 mL-doped CdSeS NCs in toluene solution are slowly added into the distilled methyl methacrylate , which contains azobisisobutyronitrile of 0.5% in weight. As a prepolymerization process, the MMA-NC solution is first heated in a flask at about 90°C for 20 min to get the suitable viscosity. It is then poured into molds and put into a desiccator at 60°C in vacuum for post polymerization. Generally, the whole process in the desiccator is conducted for more than 20 h. Optical absorption spectra from 3.10 to 1.13 eV with 1-nm step are measured on a Perkin-Elmer Lambda 900 spectrophotometer at room temperature (PerkinElmer, Waltham, MA, USA). Power X-ray diffraction (XRD) spectra are taken on a Philips X'pert diffractometer CdSeS NCs in toluene are deposited onto low-scattering quartz plates, and the solvent is evaporated. Employing a He-Cd laser (*λ* = 325 nm) as the excitation source, the low temperature photoluminescence (PL) spectroscopy is carried out to characterize the optical properties of CdSeS NCs. We performed steady-state and time-resolved luminescent studies under femtosecond laser excitation. The laser pulses are generated by a Ti: sapphire regenerative amplifier (1 KHz, Spectra Physics, Spitfire, which outputs 130 fs full width at half maximum at the central wavelength of 800 nm. The pump pulses with central wavelength of 3.10 eV are produced by high-level harmonic generation using barium boric oxide nonlinear crystal. Time-resolved luminescence is measured with ICCD (Intensified Charge Coupled Device, Andor, IStar740,) on a spectrometer (Bruker Optics 250IS/SM). The CdSeS NCs are excited by femtosecond pulses at 1.55 or 3.10 eV with a repetition rate of 83 Hz. Instrument response time *τ* is 2 ns when scattering femtosecond laser pulses are measured. All steady-state and time-resolved luminescent measurements are performed in a darkroom, and samples are placed in sealed cuvette to prevent oxidation.

## Results and discussions

### Absorption and emission properties of CdSeS NCs

**Figure 1:**
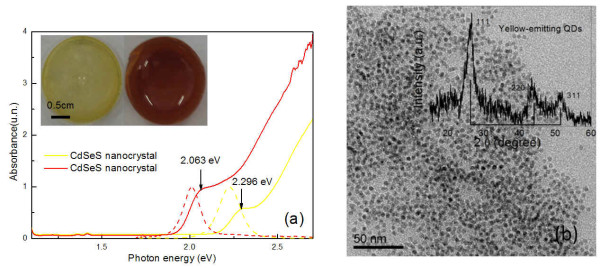
**The absorption and emission spectra and TEM image of CdSeS NCs.** (**a**) The absorption and emission spectra of yellow- and red-emitting CdSeS NCs dispersed in toluene solution, inset: the image of corresponding CdSeS NCs/PMMA matrix; (**b**) The TEM image of yellow-emitting CdSeS NCs; the size of NCs is 4.43 nm (8 %) by analysis of Image Pro-Plus software. Inset: the XRD spectrum of yellow-emitting CdSeS NCs.

**Figure 2:**
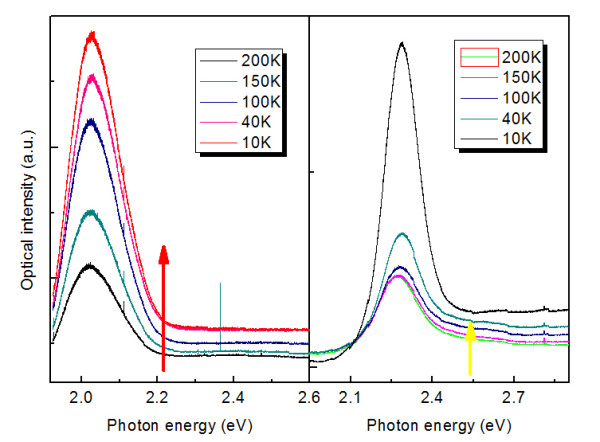
PL spectra as a function of temperature for red- and yellow-emitting CdSeS NCs in PMMA.

From Figure1 and 2, we can see the two obvious absorption peaks emerged at 2.30 and 2.06 eV for yellow- and red-emitting NCs, respectively. Luminescence spectra have obvious emission peaks at room temperature which appear similar to those of CdS and CdSe NCs. Absorption and luminescence spectra are characterized by the first excitonic peaks and the narrow FWHM, indicating fairly uniform size distribution. But the correlation between NCs size and the first absorption peak for pure CdS NCs is void presumably due to the Se dopant [16]. The energy difference ΔE=2Sℏω between the absorption and emission peaks is known as the Stokes shift can be used to describe exciton-phonon coupling. Where *ω* is the energy of the phonon coupled to the electron, and *S* is the Huang-Rhys factor. This factor is a measure of strong (large value) or weak (small value) exciton-phonon coupling. It is possible that the excitons may be coupled to more than one phonon. If, for simplicity, one assumes that the excitons are coupled to only the longitudinal optical (LO) phonon, then the factors *S* are equal at the same diameter for the NCs. Thus, one can conclude that the strength of the exciton-phonon coupling decreases with increasing Se dopant in the CdS nanocrystals. A similar trend was reported for phonon coupling and photoconductivity in CdSe_x_S_1-x_ single crystal experiment [17]. Direct transmission electron microscopy (TEM) image is used to determine the size of CdSeS NCs. By the analysis of Image Pro-Plus software, the sizes of yellow- and red-emitting NCs are 4.43 and 4.51 nm, respectively. The crystalline zinc-blende type structure is derived from powder XRD data shown in Figure1b. CdSeS NCs powder samples are indexed with the help of JCPDS file No. 100454, and the diffraction of CdSeS NCs is quite close to that of CdS and CdS NCs [18]. The three peaks in XRD diffraction patterns are not moved, indicating that Se/S molar ratio is too small to change the structure of crystal lattice. This also demonstrates that the amount of Se incorporated into CdS NCs lattice is much smaller than the amount of S precursor added to the reaction mixture. 

### Low-temperature luminescent properties of CdSeS NCs

To investigate the temperature influence on electronic structure and luminescent properties of doped NCs, the temperature dependent PL spectra of CdSeS NCs at the range of 10 to 280 K are taken and shown in Figure2. As the temperature decreases, the peak energies of PL spectra show obvious blue shifts, and the PL spectra exhibit decreased broadening and increased intensity. Here, spectral band is nonsymmetrical; a weak emission appeared at higher energy spectral range can be associated with the excitonic state of CdSeS NCs, as denoted by arrows in Figure2. The temperature dependence of the emission intensity of CdSeS NCs has been discussed, and the proposed mechanisms can reasonably explain the temperature behavior of excitonic emission observed here. Based on the theory of thermal quenching, the temperature dependence of the integrated emission intensity, *I* (*t*), can be described by [19]:

(1) $$I(t)=\frac{I_{0}}{1+Ce^{-E_{b}/KT}}$$

**Figure 3:**
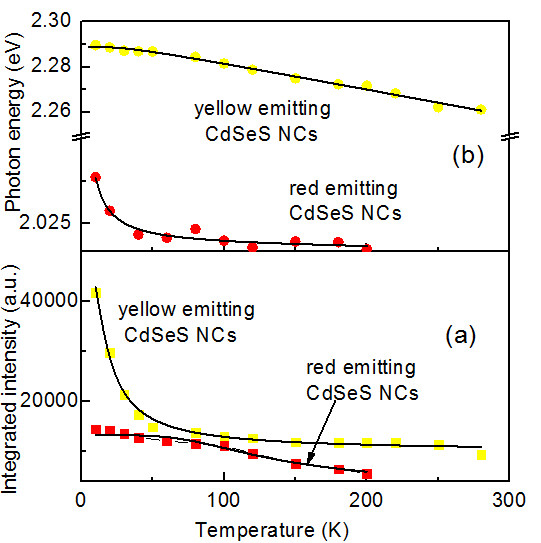
**The experimental energy gap and luminescent intensity for the sample.** (**a**) Experimental energy gap for the same sample (full squares) and best fit curve (continuous line). (**b**) Luminescent intensity has an obvious change with increasing temperature.

Here *E*_*b*_ is the activation energy (thermal quenching energy), *K* is the Boltzmann constant, *C* is a constant related to the ratio of the radiative lifetime to the nonradiative lifetime, and *I*_*0*_ is the integrated emission intensity at 0 K. The solid lines in Figure3a represent fitting curves of the intensities using Equation 1. The fitting parameters of yellow- and red- emitting NCs are shown in Table 1. It is generally accepted that the major nonradiative carrier relaxation channel in semiconductors is due to phonon quenching [20,21]. When the phonon coupling is stronger, the nonradiative rate is higher, and the luminescence is more sensitive to temperature change [22]. Moreover, the simulated results show that the phonon coupling for yellow-emitting CdSeS NCs is stronger than that of red-emitting CdSeS NCs. The deep level emission appears rapidly as T decreases. No new emission line emerges at energy above the sharp emission line as T decreases, supporting the assignment of free exciton to the sharp emission line. The good agreement between the theory and experiment suggests that the intensity increase of two emissions is mainly due to thermal quenching of phonon coupling. 

The blue shift (point lines) of the excitonic emission data is summarized in Figure3b. If we assume a constant exciton binding energy against T, the blue shift of excitonic emission line is the consequence of thermal expansion of the lattice and exciton-phonon coupling. Both effects result in the broadening of band gap in turn to the blueshift of the emission line. In general, the experimental values of energy gap of a semiconductor as a function of T are well fitted to the following empirical model based on the exciton-phonon coupling [22]:

(2) $$E_{g}(t)=E_{g}(0)-2\alpha_{B}{\left(e^{\theta /T}-1\right)}^{-1}$$

where *E*_ϵ_ (0) is the transition energy at 0 K, which is blue shifted from the bulk band gap due to quantum confinement effects; α_B_ is the strength of the electron-average phonon coupling; *θ* is the temperature corresponding to an average phonon energy. The best fitting of yellow-emitting CdSeS NCs (black lines in Figure3a) yields *E*_ϵ_ (0) = 2.289 eV, α_B_ = 5 meV, and *θ* = 85 K. The estimated average phonon temperature *θ* in the sample is lower than the LO phonon temperature (about 305 and 300 K) of cubic CdSe and CdS, indicating that acoustic phonons as well as optical phonons contribute to the red-shift of the emission energy. The *θ* value contains information about the contribution of the acoustic phonons to the blue shift of band gap emission: the smaller the *θ* value with respect to the LO phonon energy, the larger the contribution of the acoustic phonons to the band gap broadening through exciton-phonon coupling. The electron-average phonon coupling strength α_B_ is slightly smaller than that of bulk CdSe (36 meV) [23], which demonstrates that the structure of doped NCs have a more intrinsic exciton picture than pure NCs. The strength of multiple exciton-phonon coupling is enhanced with the increasing Se dopant [24]. Intensities of both the trapping and excitonic emission increased markedly at lower temperature, this effect is attributed to the suppression of phonon-coupled thermal quenching and different sensitivities of temperature dependence between trapping and excitonic state in NCs, while peak shifts at lower energy band are attributed to trapping state in low temperature spectra of CdTe NCs in the previous work [25,26]. In the doped structures, this can be understood by taking into account a competing influence by a more efficient recombination channel that causes the overall red shift of the absorption and PL peaks with comparison to that of CdS NCs with similar size. The enhancement of luminescence in higher photon energy band at lower temperature is attributed to excitonic state from CdS host NCs. The prominent luminescence is attributed to trapping state originated from the hole wave function spatially localized around the Se defect [27].

As well as we know, the ionic radius of S^2−^ is 0.17 nm, whereas that of Se^2^ is 0.18 nm. Se^2−^ ions are mostly injected into crystal lattice and substitute the site of S^2-^ ions because of good crystal matching. Several Se^2^ ions in the CdS NCs create a distribution of traps within a band gap as impurity states, which leads to a red-shifted narrow emission compared to that of pure CdS NCs. The light emission of pure CdS NCs are mostly in the blue to green spectral range; however, luminescence emission may be extended to UV and near-infrared spectral range through element-doping method. Although Se element with only a small quantity (molar ratios of Se to S are 4.0% and 10%) is in the form of CdSeS NCs, the trapping energy level due to Se impurity and surface defects is dominant on luminescence of CdSeS NCs. With the amount of Se^2−^ ions in CdSeS NCs increasing, the lowest impurity state is further lowered in energy gap. In order to further verify the assumption, we need to consider that the temperature dependence of the energy gap is usually similar to the bulk semiconductor one, except for a temperature-independent energy offset due to the quantum confinement.

### Host and composition dependent time-resolved luminescence of CdSeS NCs

**Figure 4:**
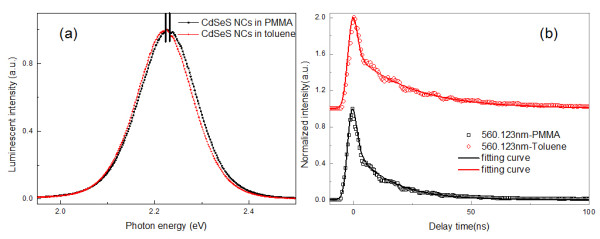
**The normalized PL spectra and PL decays of CdSeS NCs.** (**a**) Normalized PL spectra of studied CdSeS NCs dispersed in PMMA (black line), toluene (red line) (**b**) PL decays of corresponding CdSeS NCs dispersed in PMMA (black line), toluene (red line), and corresponding fitting curves.

Luminescence decay can provide additional important information for photo-induced carrier dynamics in CdSeS NCs. Luminescence of CdSeS NCs can be easily observed at 3.10 eV femtosecond laser excitation. The luminescence dynamics of yellow-emitting CdSeS NCs in different host materials (PMMA, toluene) are measured without changes of experimental conditions, and the results are shown in Figure4. Luminescence decay curve is not mono-exponential and consists of multiple exponential components. All the measured luminescence decays of emission peaks are found to be nonexponential, expressed as

(3) $$I(t)=A+\int_{0}^{t}(Be^{-(t-t')/\tau_{1}}+Ce^{-(t-t')/\tau_{2}})e^{-{\left((t-t_{0})/\tau \right)}^{2}}dt'$$

where $\tau_{1}$and $\tau_{2}$are lifetimes of short-lived excitonic states and long-lived trapping states, respectively; *t*_*0*_ is the time origin of interaction between femtosecond laser and luminescent material. *A*, *B*, and *C* are constants to be determined. *A* represents the background noise of luminescence. *B* and *C* are the relative intensity of each component within the whole normalized luminescence. The luminescence lifetime of CdSeS NCs in toluene is a little longer than that in PMMA. Furthermore, the carrier relaxation process of energy transfer between NCs easily happened in PMMA because the solid matrix provides a route, which is efficient for phonon-assisted energy transfer. The change of host materials, which corresponds to the change of dielectric coefficient and average distance between neighbor NCs, can affect energy transfer [28]. We can see the steady-state and time-resolved luminescence of yellow- and red-emitting CdSeS NCs as exhibited in Figure1a and 4. With small Se dopant incorporating into CdS, the luminescence has an enormous change with comparison to pure CdS NCs. As we know, surface atoms usually have fewer adjacent coordinate atoms and more dangling bonds and can be treated as defects as compared to the bulk atoms. These defects induce additional electronic states in the band gap as trapping state, and may also influence the spacing of the energy levels and optical properties of the NCs. [29] Peaks of trapping state created by the dopant and band-edge excitonic state are too close to each other to be distinguished [30].

Here, Se-doped NCs, in which the dopant atoms form a defect state, spatially localizes only the holes and traps electron. Hole wave function overlaps with carriers in valence band, which enhances the electronic potential and shifts up the position of valence band. With more Se incorporation, simple trapping states evolutes to a trapping energy band. One of the optical transition routes always happened between conduction band minimum (CBM) and the highest level of trapping band. When Se dopant concentration achieves a critical value, trapping energy band will merge into valance band maximum (VBM). The band gap decreases because of stronger overlap of the hole wave functions due to more Se dopant.

The biexponential fitting strongly implies the involvement of other excited states in this luminescence process for CdSeS NCs in both host materials. These energy levels are more likely to be of the surface nature. In the previous study, an intensity-dependent energy level can explain the red shift of the emission peak using infrared femtosecond laser excitation [31], where two excited states are higher-lying optical-allowed state and a lower-lying surface state with a triplet origin. Here, we show that a few atoms of Se incorporated in each dot can have a significant effect not only on the linear but also on the nonlinear properties of the entire nanocomposites [32].

In Figure4a, we can see that CdSeS NCs in PMMA has a blue shift about 2 nm with comparison to that in toluene, it is due to the oxidation of surface during the prepolymerization process. However, its luminescence has a shorter lifetime and is presumably originated from exciton-phonon coupling in NCs/PMMA. Since surface oxidation changes the position of surface-trapping state in energy gap, it is another point of view which causes a large increase in the local dielectric constant relative to the PMMA medium (ϵ_*PMMA*_ = 2.60). Toluene has a slightly lower dielectric constant (ϵ_*toluene*_ = 2.38) than PMMA and could result in an increase of the emission energy in the larger NCs [33]. PMMA as a host for exciton-phonon coupling and relatively small distance between neighboring NCs in PMMA enhance the possibility of a direct carrier transfer. It is reasonable that enhancement of excitonic state emission occurs when the host material form toluene to PMMA. Certain media, e.g., a PMMA polymer, destroy the perfection of the ligands capping, thus inducing a reduction of lifetime τ_ave_. PMMA media induces a surrounding with a relatively high dielectric constant which differs from other organic media. The lifetime of the NCs embedded in PMMA and water solution is thus scaled by a factor of ${\left[3\epsilon_{i}/(2\epsilon_{i}+\epsilon_{\mathrm{NC}})\right]}^{-2}$ from that measured in toluene solution. But simple simulations are not helpful for analysis of luminescent property because of intrinsic energy level structure. In general, trapping state emissions have longer radiative lifetimes due to lower transition probability relative to band-edge excitonic combination. The average lifetime of NCs/PMMA becomes shorter than that of dispersed in toluene. We adopt long-lived species to investigate the dependence of detection photon energy of CdSeS NCs, because faster components in biexponential decay usually reflect the excitonic recombination process of an electron and a hole. The long lifetime decay component becomes shorter from 24.2to 16.2 ns with the change of host material from dispersed in toluene to PMMA. Obviously, the lifetime (1 2 ns) of band-edge PL does not depend on detection wavelengths shown in Figure4. However, the long-lived species, which is attributed to surface or trapping state [34], rebounds to investigate the mechanism of luminescence in detail. The long lifetime component becomes longer from 12.5to 19.9 ns with the change of detection energies from 2.3to 2.1 eV. Therefore, the average PL lifetime increases steadily with increased QDs size. The wavelength dependence of the luminescence decay indicates that surface excitonic emission indicates the difference between the populations of different sizes of NCs. The larger NCs, which emit at the longer wavelength, have surface state emission with a longer lifetime. We suggest that the PMMA form a strongly coupled electronic bath that forms the environment controlling energetic fluctuations and, hence, irradiative trapping kinetics in the CdSeS NCs. The NCs/PMMA composites can be applied to fiber communication and all-optical limiting switching and electroluminescence. The work on the application of luminescent NCs/PMMA composite for brand new solid state lighting devices and optical limiting is in progress.

### Upconversion luminescence of CdSeS NCs

**Figure 5:**
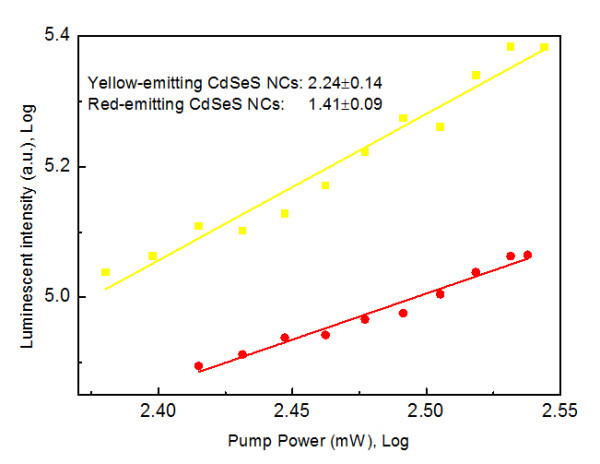
**The luminescence intensity dependence on pump power intensity of red- and yellow-emitting CdSeS NCs.** Luminescence-intensity dependence on pump power intensity of red- and yellow-emitting CdSeS NCs at 1.55 eV femtosecond laser excitation.

**Figure 6:**
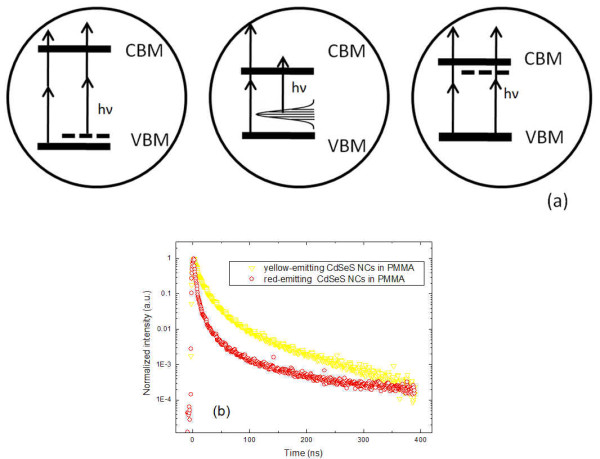
**The diagram of energy levels of trapping state for CdSeS NCs and its photoluminescence decays.** (**a**) Diagram of energy levels of trapping state for CdS NCs with increasing Se dopant. Upon the increase of Se dopant, trapping states shift up in energy, then form trapping energy band, translate to a new valence band. CBM, conduction band minimum; VBM, valance band maximum. (**b**) Normalized photoluminescence decays of studied yellow- and red-emitting CdSeS NCs embedded in PMMA (black point) and corresponding fitting curves.

The upconversion luminescence (UCL) can be easily observed using 1.55 eV femtosecond laser excitation. Power variations are adjusted using an energy attenuator and measured with a power meter. As shown in Figure5, the power-dependent study demonstrates that UCL spectra of yellow-emitting CdSeS NCs have a slope of 2.2, while that of red-emitting CdSeS NCs have a slope of 1.4. The results indicate that luminescence intensity of yellow-emitting CdSeS NCs is quadratically related pump power, but luminescence intensity of red-emitting CdSeS NCs varies abnormally with pump power. From the above discussion about low-temperature spectra of doped CdSeS NCs, we also can see the difference between the yellow-emitting and red-emitting NCs. The energy levels of trapping states appear above the top of the conduction band of CdSeS NCs. For CdSeS NCs, the traps originate from Se surface and dopant atoms, as confirmed in literature [27,35]. With increasing the amount of Se dopant, the band gap decreases because of changes of electronic structure and trapping state evolves into a series of energy level. The position of the highest level shifts up in the band energy gap. When the Se element dopant is low (for example, 4% to 10% molar ratio), the trapping state is near the top of valance band so that one 1.55 eV photon energy is not enough to excite electrons to conduction band. Thus, only two-photon process can generate an exciton. When Se concentration increases, the trapping state energy level is lifted up, the overlap between trapping state and valence band can cause energy band shrinking. At the same time, the difference between the trapping state and bottom of conduction band is less than 1.55 eV, so phonon-assisted upconversion luminescence occurred. If Se dopant continues to increase while S element becomes dopant, a series of trapping state evolves to trapping state below the top of the valence band, as shown in Figure6a. One-photon and two-photon absorption processes maybe coexist when larger amounts of Se are doped. Time-resolved luminescence of red-emitting CdSeS NC suggested that the trapping state has a shorter lifetime than yellow-emitting CdSeS NCs, shown in Figure6b. This can be attributed to the stronger exciton-phonon coupling. 

Overall, the optical properties of CdSeS NCs are found to be radically different from the binary NCs and to a certain extent from those reported for other ternary NCs. The involvement of trapping state due to anion dopant makes these materials of many potential interests in quantum dot laser and solar cell conversion.

## Conclusions

We synthesized a CdSeS NCs/PMMA composite using prepolymerization method. PMMA, a transparent material in the visible spectral range, is chosen as the embedding matrix for the NCs. The steady-state and time-resolved luminescence of Se-doped CdS NCs has been studied. The long lifetime component of CdSeS NCs in PMMA is shorter than that dispersed in toluene. We have described a model for impurity doping in semiconductor CdSeS NCs based in terms ideas of low temperature and composition dependence of luminescence. The model shows that Se dopant, which are used to control emission wavelength, play another important role by affecting energy structure. By choosing a suitable composition of NCs with special size, our model can offer a route to rational optimization of doped NCs and tailor the PL spectrum of the composite devices in the visible spectral range.

## Competing interests

The authors declare that they have no competing interests.

## Authors’ contributions

The work conducted here was collaborated with all authors. Wu designed and analyzed the luminescence and exciton-phonon coupling of NCs and drafted the manuscript. Yu carried out and analyzed the low-temperature luminescence of NCs. Gao and Ye took charge of the ultrafast laser experiment. Chang took part in all procedures and conceived the research theme as the corresponding author. All authors read and approved the final manuscript.
